# An Upper-Level Ontology for the Biomedical Domain

**DOI:** 10.1002/cfg.255

**Published:** 2003-02

**Authors:** Alexa T. McCray

**Affiliations:** National Library of Medicine Rockville Pike Bethesda MD 8600 USA

## Abstract

At the US National Library of Medicine we have developed the Unified Medical Language System (UMLS), whose goal it is to provide integrated access to a large number of biomedical resources by unifying the vocabularies that are used to access those resources. The UMLS currently interrelates some 60 controlled vocabularies in the biomedical domain. The UMLS coverage is quite extensive, including not only many concepts in clinical medicine, but also a large number of concepts applicable to the broad domain of the life sciences. In order to provide an overarching conceptual framework for all UMLS concepts, we developed an upper-level ontology, called the UMLS semantic network. The semantic network, through its 134 semantic types, provides a consistent categorization of all concepts represented in the UMLS. The 54 links between the semantic types provide the structure for the network and represent important relationships in the biomedical domain. Because of the growing number of information resources that contain genetic information, the UMLS coverage in this area is being expanded. We recently integrated the taxonomy of organisms developed by the NLM's National Center for Biotechnology Information, and we are currently working together with the developers of the Gene Ontology to integrate this resource, as well. As additional, standard, ontologies become publicly available, we expect to integrate these into the UMLS construct.


**Comparative and Functional Genomics**
*Comp Funct Genom* 2003; **4**: 80–84.
Published online in Wiley InterScience (www.interscience.wiley.com). **DOI:** 10.1002/cfg.255

**Conference Review**

### An upper-level ontology for the biomedical domain

Alexa T. McCray*
*National Library of Medicine, 8600 Rockville Pike, Bethesda, MD, USA*

**Correspondence to:
Alexa T. McCray, National Library of Medicine, 8600 Rockville Pike, Bethesda, MD, USA.
E-mail: mccray@nlm.nih.gov*

Received: 22 October 2002
Accepted: 4 December 2002

### Abstract

**At the US National Library of Medicine we have developed the Unified Medical Language System (UMLS), whose goal it is to provide integrated access to a large number of biomedical resources by unifying the vocabularies that are used to access those resources. The UMLS currently interrelates some 60 controlled vocabularies in the biomedical domain. The UMLS coverage is quite extensive, including not only many concepts in clinical medicine, but also a large number of concepts applicable to the broad domain of the life sciences. In order to provide an overarching conceptual framework for all UMLS concepts, we developed an upper-level ontology, called the UMLS semantic network. The semantic network, through its 134 semantic types, provides a consistent categorization of all concepts represented in the UMLS. The 54 links between the semantic types provide the structure for the network and represent important relationships in the biomedical domain. Because of the growing number of information resources that contain genetic information, the UMLS coverage in this area is being expanded. We recently integrated the taxonomy of organisms developed by the NLM's National Center for Biotechnology Information, and we are currently working together with the developers of the Gene Ontology to integrate this resource, as well. As additional, standard, ontologies become publicly available, we expect to integrate these into the UMLS construct. Published in 2003 by John Wiley & Sons, Ltd.**

**Keywords:** **ontologies; semantic networks; controlled vocabularies; Unified Medical Language System**

The development of an ontology is generally motivated by a particular problem that its developers are attempting to solve. In most cases, the problem itself will have a significant impact on the design, implementation and further development of the ontology. In addition, the developers' domain expertise, experience in knowledge representation methodology, and ability to maintain the ontology over a long period of time, all determine the final outcome. While there is some disagreement about what qualifies as an ontology, most agree that an ontology is a representation of a domain of interest, which, at a minimum, involves naming the basic concepts in that domain [6]. Such a definition would allow a simple list of terms to be an ontology. A more formal definition of an ontology would, however, require that the relationships between and among these concepts be made explicit. These relationships may be taxonomic links, resulting in hierarchically organized concepts, or they may include additional non-hierarchical relationships, together with some constraints on how these relationships are to be interpreted [2,8,9].

The purpose to which the ontology will be put determines the nature and type of ontology that is created. While a simple list of controlled terms can be sufficient for indexing documents and other datasets, even here some complexity, e.g. in the form of synonyms, is often added once the terminology is put to use. Placing the concepts in a hierarchy provides another level of complexity,

This article is a US Government work and is in the public domain in the USA.

but it also allows for greater flexibility in searching, making it possible for a user to formulate a query that asks for items indexed not only under a particular concept but also, for example, under all the descendants of that concept in the hierarchy. This may be all that is ever needed for information-retrieval purposes, but if the ontology is intended to be used in an application that requires reasoning in a knowledge-based application, such as a decision support system, then a richer set of relationships between concepts will be needed.

At the US National Library of Medicine, we have developed a system whose goal it is to provide integrated access to a large number of biomedical resources by unifying the domain vocabularies that are used to access those resources [4,10]. The Unified Medical Language System (UMLS) project currently interrelates some 60 controlled vocabularies in the biomedical domain. The vocabularies vary in nature, size and scope and have been created for widely differing purposes. Some consist of a list of a few hundred terms, while others contain tens of thousands of interrelated concepts. Some vocabularies have been created for document retrieval systems, others for coding medical records for billing and administrative purposes, and yet others have been created for use in medical decision support systems. Some are highly specific to a particular medical specialty, such as the National Cancer Institute's Physician Data Query (PDQ) system, the psychiatrists' Diagnostic and Statistical Manual of Mental Disorders (DSM-III and DSM-IV), and the nurses' Classification of Nursing Diagnoses. Others are targeted to particular fields of study, such as the University of Washington's anatomy terminology. Several large vocabularies are quite broad and deep in their scope, including the Systematized Nomenclature of Medicine and the Medical Subject Headings (MeSH). The Metathesaurus contains almost 900 000 concepts drawn from its multiple vocabularies. Its coverage is quite extensive, including not only many concepts in clinical medicine but also a large number of concepts applicable to the broad domain of the life sciences, e.g. MeSH, a thesaurus of some 19 000 concepts in the biomedical domain, has many concepts in the areas of anatomy, biology, physiology, organisms, diseases and chemicals. It also has a large number of concepts in molecular biology and genetics, e.g. cytogenetics, medical genetics, genetic recombination and mutations.

When a new vocabulary is added to the UMLS, its constituent terms are linked whenever possible to existing Metathesaurus concepts. Thus, if a new clinical vocabulary has, for example, the disease name 'lymphogenous leukemia' and if the concept 'lymphocytic leukemia' already exists in the Metathesaurus, then the new name is added to the existing concept as a synonym. Similarly, if the new vocabulary has the term 'acute lymphocytic leukemia', and this concept does not already exist in the Metathesaurus, then a new concept is formed, and it is linked to the most closely related UMLS concept. In this case, since 'acute lymphocytic leukemia' is not a synonym of 'lymphocytic leukemia', it is linked to the latter concept as a narrower concept.

Early in the UMLS project, in order to provide an overarching conceptual framework for all UMLS concepts, we developed an upper-level ontology, which we call the UMLS semantic network. Semantic networks have been created and used in artificial intelligence applications for some time [3,7], and a number of groups are currently collaborating in the development of standards for upper-level ontologies encompassing a variety of domains [5]. Each UMLS concept is assigned one or more semantic types from the semantic network. The internal structure of the constituent vocabulary is maintained, so that it is always possible to view the original contexts in which a particular concept has appeared. The role of the semantic network is to provide the higher-level framework in which all concepts are given a consistent and semantically coherent representation.

The UMLS semantic network currently consists of 134 semantic types and 54 relationships. The network is defined at the highest level by two hierarchies, one for entities and another for events. Each semantic type is linked to its parent by the 'is_a' link, e.g. 'Human' is a leaf node in the 'Entity' hierarchy. Traversing the 'is_a' links from 'Human' to 'Entity' allows the following statements: a human is a mammal, which is a vertebrate; a vertebrate is an animal, which is an organism; an organism is a physical object, which is an entity. In addition to the definitional power of the network itself, each semantic type is given a textual definition. The definition is helpful for assigning, as well as interpreting, semantic types linked to Metathesaurus concepts, e.g. the definition for the semantic type 'Mammal' is 'a vertebrate having a constant body temperature and characterized by the presence of hair, mammary glands and sweat glands'. Accompanying each definition are examples of instances of that type found in the Metathesaurus. In this case, some instances are 'bears', 'Guernsey cow', '*Rattus norvegicus*', and 'whales'. In some cases, usage notes are included with the definitional information and these serve as clear guidelines for semantic type assignment by UMLS curators, e.g. most drugs can be viewed from the perspective of both their therapeutic or functional activities and their underlying structural properties. Usage notes for drug semantic type assignment, therefore, indicate that both a functional and a structural semantic type should be chosen.

The semantic network is further defined by a set of associative relationships, which themselves form a hierarchy. The top-level associative relationships are 'physically related to', 'spatially related to', 'functionally related to', 'temporally related to', and 'conceptually related to'. Table 1 shows the complete set of relationships currently available in the UMLS semantic network.

A typical assertion might be 'Pharmacologic Substance treats Disease or Syndrome', where 'Pharmacologic Substance' and 'Disease or Syndrome' are semantic types, and 'treats' is one of the relationships that obtains between them. Figure 1 shows a portion of the UMLS semantic network, illustrating some of the relationships that interrelate the semantic types.

Note that the associative relationships are stated at the highest possible level, and are inherited by the descendants of those types, e.g. because biological function is a process of an organism, it is also a process of an animal, a vertebrate, a mammal and a human. Analogously, since a genetic function is a molecular, physiologic, and biologic function, it is also a process of an organism, and therefore also of a human.

Because of the growing number of information resources that contain genetic information, it has become clear that the UMLS coverage in this area needs to be extended. Some of the UMLS vocabularies contain terminology at the cellular and molecular level, but none has been created specifically for genetic resources. As a first step in extending the coverage of the UMLS with concepts relevant to the genomic domain, we recently integrated the taxonomy of organisms developed

**Table 1.** UMLS semantic network relationships

| |
|---|
| is_a |
| associated_with |
|   physically_related_to |
|     part_of |
|     consists_of |
|     contains |
|     connected_to |
|     interconnects |
|     branch_of |
|     tributary_of |
|     ingredient_of |
|   spatially_related_to |
|     location_of |
|     adjacent_to |
|     surrounds |
|     traverses |
|   functionally_related_to |
|     affects |
|       manages |
|       treats |
|       disrupts |
|       complicates |
|       interacts_with |
|       prevents |
|     brings_about |
|       produces |
|       causes |
|     performs |
|       carries_out |
|       exhibits |
|       practices |
|     occurs_in |
|       process_of |
|     uses |
|     manifestation_of |
|     indicates |
|     result_of |
|   temporally_related_to |
|     co occurs_with |
|     precedes |
|   conceptually_related_to |
|     evaluation_of |
|     degree_of |
|     analyzes |
|       assesses_effect_of |
|     measurement_of |
|     measures |
|     diagnoses |
|     property_of |
|     derivative_of |
|     developmental_form_of |
|     method_of |
|     conceptual_part_of |
|     issue_in |

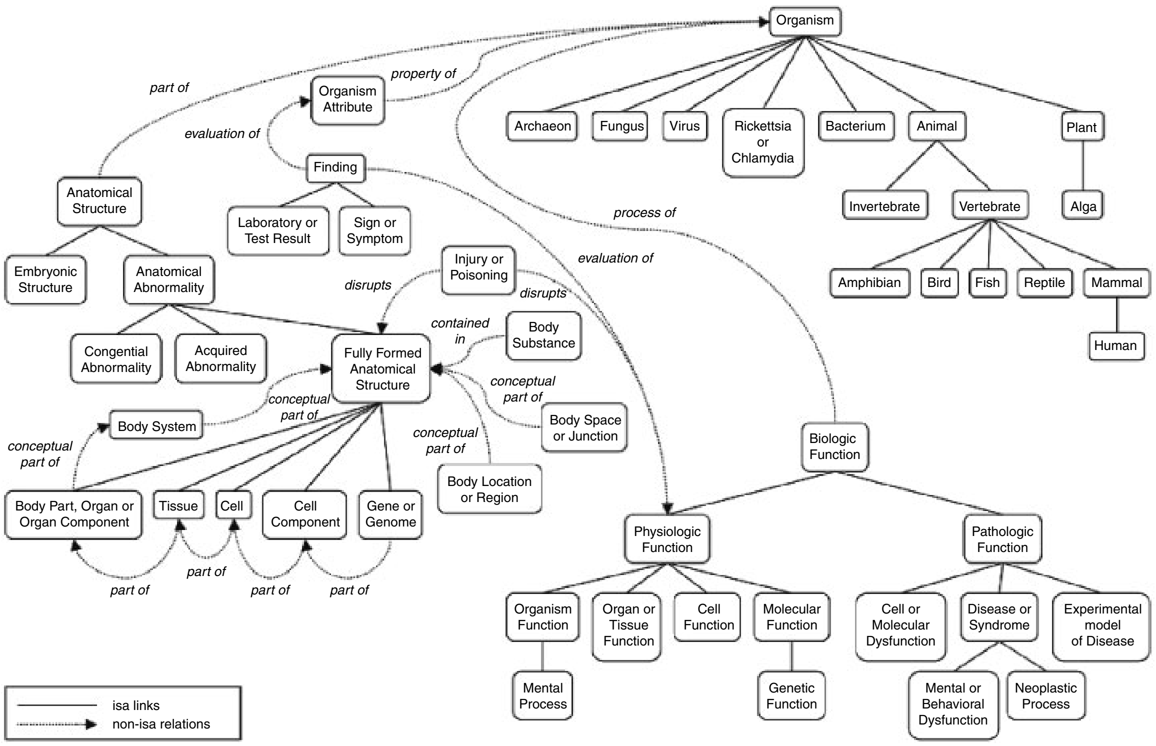

**Figure 1.** A portion of the UMLS semantic network by the NLM's National Center for Biotechnology Information (NCBI). The NCBI taxonomy is a rapidly evolving taxonomy of organisms, incorporating both phylogenetic and taxonomic knowledge from a variety of sources [12]. The taxonomy currently contains more than 100 000 organism names, including archaea, bacteria, eukaryota and viruses. The coverage of the taxonomy is determined by the names for organisms whose sequences have been made public in a variety of sequence databases, including GenBank, European Molecular Biology Laboratory (EMBL), the SWISS-PROT protein sequence database and Protein Information Resource (PIR).

We are working together with the developers of the Gene Ontology (GO) to integrate this ontology with the UMLS [1]. Our initial algorithmic mapping indicates that the three GO components, molecular function, biological process and cellular component, map unevenly to existing UMLS concepts. The greatest number of concepts mapped to the UMLS is found in the molecular function component. About 43% of the GO molecular function component terms mapped to UMLS concepts, and about 35% of the cellular components mapped. A relatively small number of the GO biological processes were found (about 5%). The algorithmic mappings are currently being reviewed and modified by a GO curator in collaboration with UMLS curators at the NLM. As part of this effort, we are reviewing the semantic network for its coverage of the genetic domain. Semantic types and relationships that will be useful include, for example, 'Cell Component', 'Biologically Active Substance', 'Genetic Function', 'Nucleotide Sequence', 'Enzyme', 'Molecular Biology Research Technique', 'part_of', 'process_of', 'interacts_with', but it is likely that more will be needed.

Knowledge in genomics continues to evolve at a pace that could not have been imagined even a decade ago, and there are ongoing efforts in the genomics community to develop standard nomenclatures for this domain [11]. As additional, standard, ontologies become publicly available, we expect to integrate these into the UMLS construct, which is regularly updated and readily available to the community. As our understanding of biological processes, particularly at the cellular and molecular level, continues to increase, so, too, will our understanding of the pathogenesis of disease. Genomic databases provide the raw data for making these discoveries. Results of these investigations are published in the scientific literature and, in time, this knowledge will be stored in a variety of clinical information systems. At every step, standard ontologies play an important role. To the extent that we, as a community, are able to successfully develop, maintain, integrate and use these ontologies, we will be making a contribution to scientific progress in this important domain.

### References

1. The Gene Ontology Consortium. 2001. Creating the gene ontology resource: design and implementation. *Genome Res* **11**(8): 1425–1433.
2. Guarino N. 1995. Formal ontology, conceptual analysis and knowledge representation. *Int J Hum Comput St* **43**: 625–640.
3. Lehmann F. 1992. *Semantic Networks in Artificial Intelligence*. Pergamon: Tarrytown, New York.
4. McCray AT, Nelson S. 1995. The representation of meaning in the UMLS. *Methods Inf Med* **34**(1–2): 193–201.
5. Niles I, Pease A. 2001. Towards a standard upper ontology. In *Formal Ontologies in Information Systems*, Welty C, Smith B (eds). ACM Press: New York; 2–9.
6. Poli R. 1996. Ontology for knowledge representation. In *Knowledge Organization and Change*, Green R (ed.). Indeks: Frankfurt; 313–319.
7. Quillian M. 1968. Semantic memory. In *Semantic Information Processing*, Minsky M (ed.). MIT Press: Cambridge, MA; 227–270.
8. Schulze-Kremer S. 1998. Ontologies for molecular biology. In *Pac Symp Biocomput* 695–706.
9. Stevens R, Goble CA, Bechhofer S. 2000. Ontology-based knowledge representation for bioinformatics. *Brief Bioinform* **1**(4): 398–414.
10. Unified Medical Language System (UMLS): **http://umlsinfo.nlm.nih.gov/**
11. Wain HM, Bruford EA, Lovering RC, *et al*. 2002. Guidelines for human gene nomenclature. *Genomics* **79**(40): 464–470.
12. Wheeler DL, Chappey C, Lash AE, *et al*. 2000. Database resources of the National Center for Biotechnology Information. *Nucleic Acids Res* **28**(1): 10–14.
